# Supplementary material for: Molecularly targeted photothermal ablation improves tumor specificity and immune modulation in a rat model of hepatocellular carcinoma
Source: Commun Biol. 2020 Dec 17;3:783. doi: 10.1038/s42003-020-01522-y (PMC7746712; doi:10.1038/s42003-020-01522-y)

Molecularly targeted photothermal ablation for local control and immunologic modulation of hepatocellular carcinoma

Nina Muñoz[1], Crystal Dupuis[1], Malea Williams[1], Katherine Dixon[1], Amanda McWatters[1], Rony Avritscher[1], Richard Bouchard[2], Ahmed Kaseb[3], Kyle Schachtschneider[4], Arvind Rao[5], Rahul A. Sheth[1]

[1]Department of Interventional Radiology, [2]Department of Imaging Physics, [3]Department of Gastrointestinal Medical Oncology, The University of Texas MD Anderson Cancer Center, Houston, TX, [4]Department of Radiology, University of Illinois at Chicago, Chicago, IL, [5]Department of Computational Medicine & Bioinformatics, University of Michigan, Ann Arbor, MI

Supplemental Table

Table S1. NanoString DSP protein markers

aSMA	B7H3	Beta Catenin	CD11b
CD11c	CD127	CD14	CD163
CD19	CD27	CD34	CD3e
CD4	CD40	CD40L	CD45
CD68	CD73	CD8a	CTLA-4
F4/80	Fibronectin	FoxP3	GAPDH
GITR	GZMB	Histone H3	ICOS
Ki67	Lag3	Ly-6G	MHC II
Mouse IgG1	OX40L	PanCK	PD-1
PD-L1	Rabbit IgG1	Rat IgG2a kappa	Rat IgG2b
S100A9	S100B	S6	Stat3
TGF beta 1	TIM3	Vista	

Supplemental Figure

Figure S1. Flow cytometry dot plots. A, Gating strategy for the evaluation of CD3+/CD8+ T cells. B, There was a significant increase in the percentage of CD3+/CD8+ following MPTA and RFA relative to sham surgery.

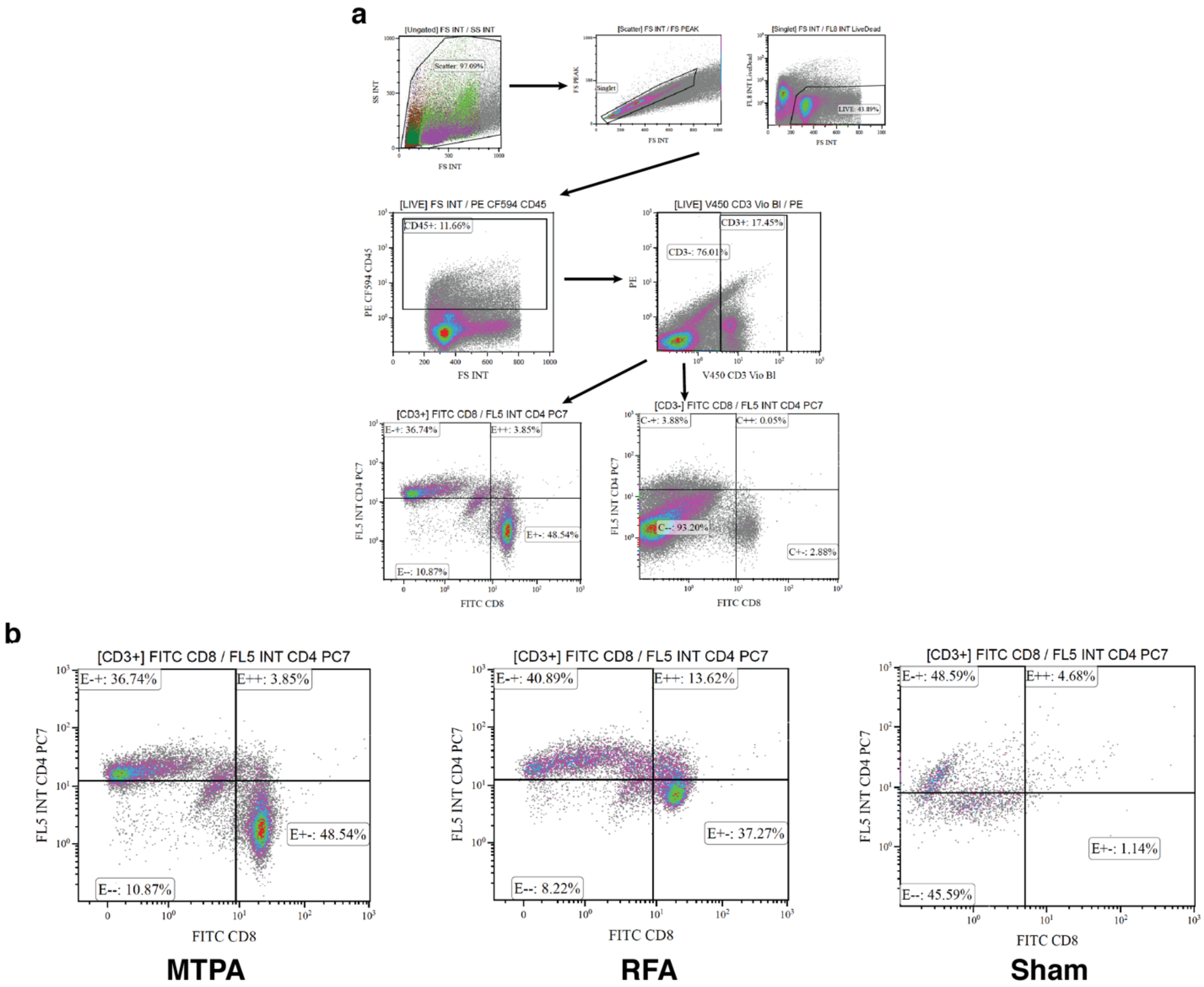

Supplement: Supplementary file 1 — Supplementary Information [file 42003_2020_1522_MOESM1_ESM.pdf]
