# Supplementary material for: Molecularly targeted photothermal ablation improves tumor specificity and immune modulation in a rat model of hepatocellular carcinoma
Source: Commun Biol. 2020 Dec 17;3:783. doi: 10.1038/s42003-020-01522-y (PMC7746712; doi:10.1038/s42003-020-01522-y)
Supplement: Supplementary file 2 — Reporting Summary [file 42003_2020_1522_MOESM2_ESM.pdf]

nature research

Corresponding author(s): Rahul Sheth

Last updated by author(s): Jun 9, 2020

Reporting Summary

Nature Research wishes to improve the reproducibility of the work that we publish. This form provides structure for consistency and transparency in reporting. For further information on Nature Research policies, see our Editorial Policies and the Editorial Policy Checklist.

Statistics

For all statistical analyses, confirm that the following items are present in the figure legend, table legend, main text, or Methods section.

n/a	Confirmed	
☐	☒	The exact sample size (*n*) for each experimental group/condition, given as a discrete number and unit of measurement
☐	☒	A statement on whether measurements were taken from distinct samples or whether the same sample was measured repeatedly
☐	☒	The statistical test(s) used AND whether they are one- or two-sided<br>*Only common tests should be described solely by name; describe more complex techniques in the Methods section.*
☒	☐	A description of all covariates tested
☐	☒	A description of any assumptions or corrections, such as tests of normality and adjustment for multiple comparisons
☐	☒	A full description of the statistical parameters including central tendency (e.g. means) or other basic estimates (e.g. regression coefficient) AND variation (e.g. standard deviation) or associated estimates of uncertainty (e.g. confidence intervals)
☐	☒	For null hypothesis testing, the test statistic (e.g. *F*, *t*, *r*) with confidence intervals, effect sizes, degrees of freedom and *P* value noted<br>*Give P values as exact values whenever suitable.*
☒	☐	For Bayesian analysis, information on the choice of priors and Markov chain Monte Carlo settings
☒	☐	For hierarchical and complex designs, identification of the appropriate level for tests and full reporting of outcomes
☒	☐	Estimates of effect sizes (e.g. Cohen's *d*, Pearson's *r*), indicating how they were calculated

Our web collection on statistics for biologists contains articles on many of the points above.

Software and code

Policy information about availability of computer code

Data collection	Gallios software was used to acquire flow cytometry data. No custom code was used to collect or analyse data.
Data analysis	Statistical analysis was performed in R (The R Foundation, version 3.5.1) using pre-existing libraries. Flow cytometry data was analysed using FlowJo (v10.6.2).

For manuscripts utilizing custom algorithms or software that are central to the research but not yet described in published literature, software must be made available to editors and reviewers. We strongly encourage code deposition in a community repository (e.g. GitHub). See the Nature Research guidelines for submitting code & software for further information.

Data

Policy information about availability of data

All manuscripts must include a data availability statement. This statement should provide the following information, where applicable:

- Accession codes, unique identifiers, or web links for publicly available datasets
- A list of figures that have associated raw data
- A description of any restrictions on data availability

The authors declare that all data supporting the findings of this study are available within the paper and as Supplementary Data. Genomic data will be deposited in a public repository, and accession codes will be provided prior to publication.

Field-specific reporting

Please select the one below that is the best fit for your research. If you are not sure, read the appropriate sections before making your selection.

☒ Life sciences ☐ Behavioural & social sciences ☐ Ecological, evolutionary & environmental sciences

For a reference copy of the document with all sections, see nature.com/documents/nr-reporting-summary-flat.pdf

Life sciences study design

All studies must disclose on these points even when the disclosure is negative.

Sample size	Sample size was determined from pilot experiments and/or experiments from the literature to obtain statistically significant data.
Data exclusions	No animals were excluded from the study.
Replication	Experiments were repeated at least once, or data were complied from two independent experiments, unless otherwise stated in the manuscript.
Randomization	Animals were acquired from the institutional colony, numbered, and then randomized.
Blinding	For tumor measurements, a team member blinded to the treatment group performed the measurements.

Reporting for specific materials, systems and methods

We require information from authors about some types of materials, experimental systems and methods used in many studies. Here, indicate whether each material, system or method listed is relevant to your study. If you are not sure if a list item applies to your research, read the appropriate section before selecting a response.

Materials & experimental systems

n/a	Involved in the study	
☐	☒	Antibodies
☐	☒	Eukaryotic cell lines
☒	☐	Palaeontology and archaeology
☐	☒	Animals and other organisms
☒	☐	Human research participants
☒	☐	Clinical data
☒	☐	Dual use research of concern

Methods

n/a	Involved in the study	
☒	☐	ChIP-seq
☐	☒	Flow cytometry
☒	☐	MRI-based neuroimaging

Antibodies

Antibodies used	CD45-PE/Dazzle594 (BioLegend cat# 202223), CD3-VioBlue (Miltenyi cat# 130-102-677), CD8-FITC (BD Pharmingen cat# 554856), and CD4-PE/Cy7 (BD Pharmingen cat# 561578).
Validation	All antibodies were purchased from the vendors above contain validation data as provided by the vendor.

Eukaryotic cell lines

Policy information about cell lines

Cell line source(s)	McArdle RH-7777 hepatoma cells were purchased from ATCC.
Authentication	Cell line authentication was performed by the institution's Characterized Cell Line Core Facility using short tandem repeat (STR) fingerprinting.
Mycoplasma contamination	All cell lines tested negative for mycoplasma infection.
Commonly misidentified lines (See ICLAC register)	No commonly misidentified cell lines were used,.

Animals and other organisms

Policy information about studies involving animals; ARRIVE guidelines recommended for reporting animal research

Laboratory animals	8-12 week male and female Buffalo rats were acquired from our institution's colony.
Wild animals	This study did not involve wild animals.
Field-collected samples	This study did not involve samples collected from the field.
Ethics oversight	All animal experiments were approved by The University of Texas MD Anderson Cancer Center's Institution Animal Care and Use Committee (IACUC).

Note that full information on the approval of the study protocol must also be provided in the manuscript.

Flow Cytometry

Plots

Confirm that:

☒ The axis labels state the marker and fluorochrome used (e.g. CD4-FITC).

☒ The axis scales are clearly visible. Include numbers along axes only for bottom left plot of group (a 'group' is an analysis of identical markers).

☒ All plots are contour plots with outliers or pseudocolor plots.

☒ A numerical value for number of cells or percentage (with statistics) is provided.

Methodology

Sample preparation	Single cell suspension creation and flow cytometry analysis<br>The tumors were dissected and cut into small pieces with a scalpel. The tissue fragments were subject to enzymatic digestion in 3 volumes of PBS containing DNAse I 1mg/mL (Roche cat# 11284932001), Collagenase D 1mg/mL (Roche cat# 11088882001) and Dispase II 2.4U/mL (Roche cat# 4942078001). After incubation at 37 C for 30min with gentle stirring, the lysate was washed with PBS+2% FBS and strained through a 40µM nylon mesh. The red blood cells were then lysed with ammonium chloride buffer (Miltenyi cat #130-094-183). The cell suspension was washed and resuspended in PBS+2%FBS before determining the total number of cells obtained as well as their viability using Trypan blue.<br>The leukocytes (i.e. CD45+ cells) were then purified using CD45 MicroBeads (Miltenyi cat#130-109-682) following the manufacturer's indications. The purified cells were resuspended in ice-cold PBS+2%FBS, and the total number and viability was determined with an automatic cell counter (Countess II, Thermo Fisher).
Instrument	Gallios 561 flow cytometer
Software	Gallios software
Cell population abundance	N/A
Gating strategy	Gating strategies are depicted in the Supplementary Data.

☒ Tick this box to confirm that a figure exemplifying the gating strategy is provided in the Supplementary Information.
